# Solid oxide fuel cell (SOFC) control strategy enhancement by adaptive neuro-fuzzy inference system (ANFIS)

**DOI:** 10.1038/s41598-024-62414-3

**Published:** 2024-08-02

**Authors:** Sameh Ramadan, Mostafa Al-Gabalaw, Mohamed EL-Shimy, Adel Emarah

**Affiliations:** 1 Electric Power and Machines Department, Ain Shams University, Cairo, Egypt; 2 Electric Power Engineering Department, Suez University, Suez, Egypt

**Keywords:** Electrical and electronic engineering, Energy grids and networks

## Abstract

Global warming is a vital problem that many researchers tried to solve with so many solutions such as: reducing electricity production with conventional generators by using renewable energy resources and using hydrogen as an alternative to fossil fuels. The universal Economic crisis came across to cut off certain quantities at scheduled times. This, directly, affects renewable energy sources connected to the grid due to voltage and frequency variations. To solve this dilemma, in this paper, the grid-connected solid oxide fuel cell (SOFC) model which is fed by green hydrogen to produce AC power, is developed by an Adaptive neuro-fuzzy inference system (ANFIS). It is one of the artificial intelligence applications to improve grid-connected SOFC dynamic response. ANFIS is mathematically presented and simulated using MATLAB/SIMULINK. The results show that the ANFIS controller has succeeded in enhancing most of the desired control and operation signals.

## Introduction

One of the most dangerous lives threatening on earth, is global warming. The normal sun- heat cycle is to be present in the atmosphere during daylight as well as disappearing at night. Unfortunately, greenhouse gases (GHG) such as carbon dioxide (Co_2_) and methane (CH_4_) Can trap the sun's heat preventing it from leaving the atmosphere during the night which disturbs this natural cycle causing global warming^1^.

As Known, GHG is mainly a result of burning fossil fuels for many purposes. Mainly generating electrical power by conventional generators and internal combustion engines (ICE) in vehicles is the most fatal causes. Nowadays, the global urge is to find alternative technologies of conventional power generators and ICE with zero-carbon emissions. Hydrogen can play a key role as an alternative fuel since it is a zero-carbon energy carrier which nominate it to be the main fuel in the future^2^.

Another option is the renewable energy sources (RES) that produce electrical energy with zero or low carbon emissions. The increasing reliance on RES has contributed to the increase in DC and AC microgrids and hybrid DC/AC microgrids which are formed of DC microgrids connected to AC microgrids through the interlinking converter (ILC). As the DC part of the hybrid microgrids is connected to the grid through an inverter or bidirectional AC/DC converter, the mode of operation is called online, or on-grid mode and the connecting point is called the point of common coupling (PCC). Nevertheless, when the DC part is not connected to the PCC, the mode of operation is called isolated mode or off-grid mode. In grid-connected mode, the main grid is responsible for the voltage and frequency stability on the AC side.

Therefore, many control strategies are developed to regulate the voltage on the DC side and manage the power flow from side to side considering the bidirectional AC/DC converter-rated power. In^3^, DC microgrids are connected to the grid and controlled by a control strategy based on measuring the amount of power injected from the DC side into the main grid. If the power exchanged is greater than the converter-rated power, the converter is turned from a U-Q control strategy to a PQ control strategy. However, if the power exchanged is less than the converter-rated power, the converter can be controlled by the U-Q control strategy or PQ control strategy. These two control strategies are demonstrated in detail in^3^.

In^4^, DC microgrids are connected to the grid through an inverter which is controlled by two cascaded loops, the inner loop and the outer loop. The inner loop role is controlling the grid current and reducing the harmonics. The outer loop role is controlling the DC voltage. These two loops form the inverter's general control structure and it can be controlled by the many control types such as:

- Linear Controllers e.g. PID controllers, State-Feedback Controllers, and, Predictive Controllers.
- Non-linear Controllers e.g. Sliding Mode Controller (SMC) and Hysteresis Controllers.
- Intelligent controllers e.g. Neural networks (NN) controllers and fuzzy logic (FL) controllers.

Controversially, the main disadvantage of RES is its intermittent nature^5^. The load profile system cannot be met by RES alone. Therefore, many researchers concluded that the best way to benefit from hydrogen and renewable energy in a system that brings them together.

The system for converting hydrogen into electrical power using RES is fully explained in^6^. This system was named Power to Hydrogen to Power (P2H2P) due to the stages of electricity production.

**Figure 1:**
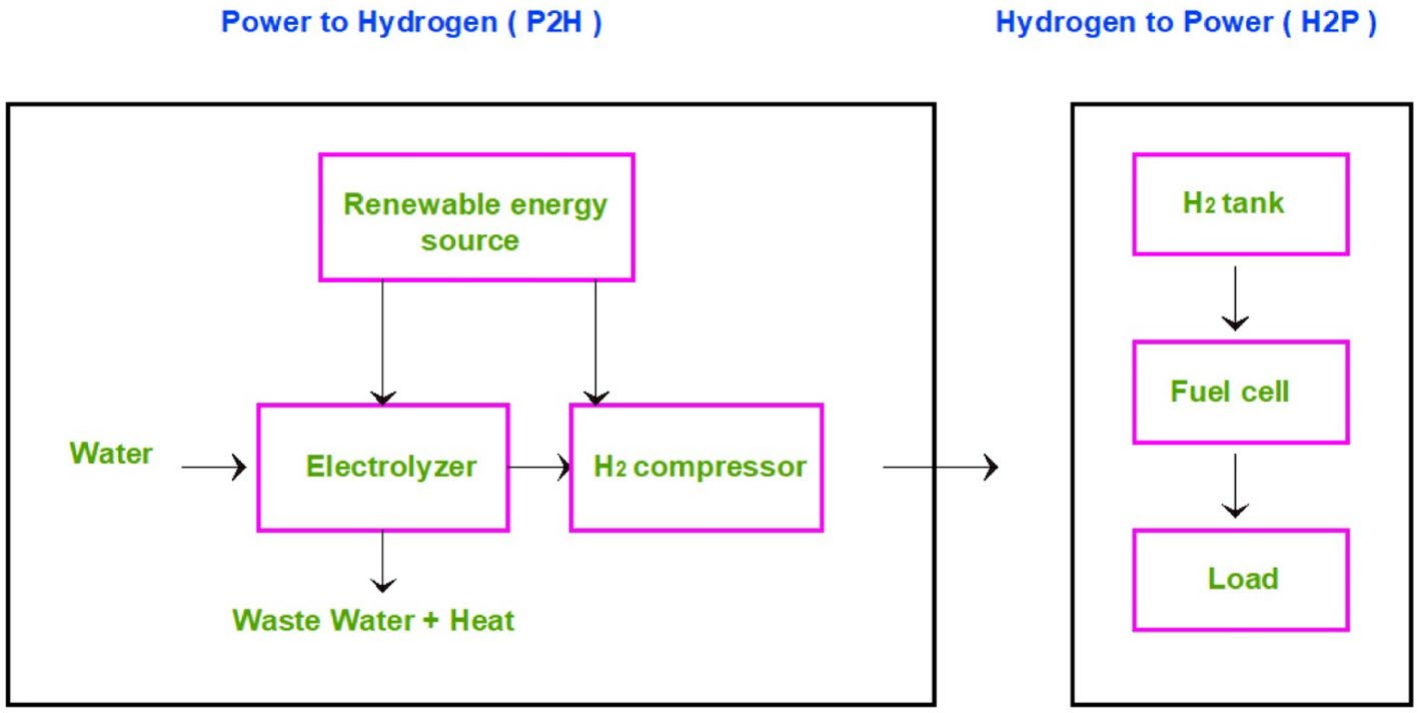
Power to hydrogen (P2H) and hydrogen to power (H2P) system.

As shown in Fig. 1, P2Hrefers to using renewable energy to produce hydrogen through the electrolyzer. The electrolyzer separates water (H_2_O) into hydrogen and oxygen by basing electrical current through the water solution in addition to the catalyst. The Water amount that did not participate in the chemical reaction and did not decompose into hydrogen and oxygen is considered wastewater.

H2P refers to using hydrogen to produce DC power through a fuel cell (FC). There are many types of FC e.g. alkaline fuel cells (AFC), molten-carbonate fuel cells (MCFC), and, solid oxide fuel cells (SOFC). The SOFC operates at very high temperatures so it is convenient to be used in power plants and it can be fueled by natural gas or hydrocarbon-based fuel or hydrogen. The aforementioned advantage is the main reason to focus on SOFC in this paper^7^.

The FC consists of an anode and cathode and the electrolyte between them. The hydrogen flows into the anode. Due to the catalyst, the hydrogen molecules are divided into protons and electrons. The hydrogen electrons combined with the oxygen electrons and based through electrical load forming an electrical current. Then returns through the cathode to combine with provided oxygen. And continue through the electrolyte to separate protons from electrons and repeat the electrochemical process as long as hydrogen and oxygen are provided to the fuel cell. The form of the reaction is:

1 $${2H}_{2}\left(gas\right)+ {O}_{2}\left(gas\right)\to {2H}_{2}O+(Electiricity+Heat)$$

Using P2H2P, hydrogen is produced without any carbon emissions, which is why it is considered a method of producing green hydrogen. After finding a system to optimally use hydrogen and renewable energy, interest became in how to benefit from and control it.

Many researchers have been fascinated by the dynamic performance of the SOFC. In^8^, using MATLAB/SIMULINK, the SOFC dynamic performance was studied by connecting it directly to the DC load and changing this load power by several steps. Then, the SOFC is connected to AC load through an inverter and the load power is changed in steps. After that, the interest in enhancing the grid-connected SOFC dynamic performance increased, in^9^, the grid-connected SOFC performance was enhanced using a PI controller by controlling the SOFC utilization factor ${U}_{f}$ i.e. the ratio of hydrogen flow rate that participates in the chemical reaction within the SOFC to the entire hydrogen flow rate that enters the SOFC.

In^10^, the grid-connected SOFC is enhanced using PI-based two control strategies which are the constant voltage control strategy and the constant ${U}_{f}$ control strategy, and the results show that the constant ${U}_{f}$ control strategy succeeded in enhancing the durability of SOFC.

In^11^ and^12^, there is a complete explanation of the grid-connected SOFC modeling and an explanation of two different strategies for controlling it and testing its dynamic response. The two strategies are the constant power control strategy and the constant current control strategy.

In^13^, a comparison was made between the two mentioned control strategies, and the resulted in that the constant current control strategy was superior to the constant power control strategy.

One of the most crucial problems facing the electrical networks in Egypt and many other countries worldwide now, is the lack of fuel necessary to generate energy for all the loads connected to the grid due to the economic crisis. In Egypt, the Ministry of Electricity is, so far, facing this problem by reducing loads through disconnecting them at fixed, scheduled times to distribute them among all consumers.

This is currently the case, but in the future, the most important proposed solution to solve this problem is to increase the percentage of renewable energy penetration that helps the network to cover the loads aside with reducing the percentage of GHG in the atmosphere. Until this happens, load disconnecting will affect renewable energy systems currently connected to the grid.

This research is investigating the possibility of using artificial intelligence (AI) to improve the dynamic response of SOFC connected to the network and controlled by a constant current control strategy when loads are reduced by a certain percentage at a specific time. The adaptive neuro-fuzzy inference system (ANFIS), which is one of the main applications of AI, is explained and represented mathematically. Then, it will also be used and simulated using MATLAB/SIMULINK to study the effect of SOFC grid-connected using ANFIS.

## ANFIS technique

Artificial intelligence is one of the most important techniques in science society. AI’s main goal is the regression of the human role in order to decrease the percentage of human error by training the machine to mimic human brain functions such as recognizing, thinking, deciding, and, solving problems^14^. This transforms machines into gaining experience and using it to improve their performance without previous programming. This process is called machine learning (ML)^14^. In the human body, neurons are responsible for the process of communicating with the brain, and by simulating this with AI, artificial neural networks (ANN) became one of the common algorithms used in ML. Human brain uses another technique to find out a problem’s solution by guessing or speculating based on previous experience. Machine language is based on only two signals which are 0, and 1^15^. AI can teach computers to think uncertainly and make decisions using fuzzy sets that consist of contentious values^16^. In 1965, Lotfi Zadeh invented a fuzzy logic system i.e. a system using previous experience to solve a problem^17^. Adaptive neuro-fuzzy inference system (ANFIS) is an AI technique for teaching ANN how to perform their function using a fuzzy inference system (FIS)^18^.

### Fuzzy logic inference systems

Boolean logic is known as crisp logic. It is about absolute values 0 or 1. Zero for false and one for true and there cannot be any value in-between. Fuzzy logic is a superset of the values in-between true and false. Each value in the fuzzy set is a partial of the absolute true value. The degree how much this value is close to true or false is called membership value^15^. As mentioned in^18^ fuzzy logic model consists of four parts:

- **Fuzzification**: It is a process of converting input crisp values to fuzzy values by assigning a membership value to each crisp value.
- **Rule functions**: They are rules put by the experts in the IF–THEN form to design the system's controllers.
- **Inference method**: There are several methods to manipulate the fuzzified values and compute the fuzzy output such as:
- Mamdani method
- Larsen method
- Sugeno method
- **Defuzzification**: It is a process of converting output fuzzy values to crisp values by defuzzification function which differs from each other based on the inference method.

The Sugeno method is the inference method that is used in the ANFIS.

### Artificial neural networks

**Figure 2:**
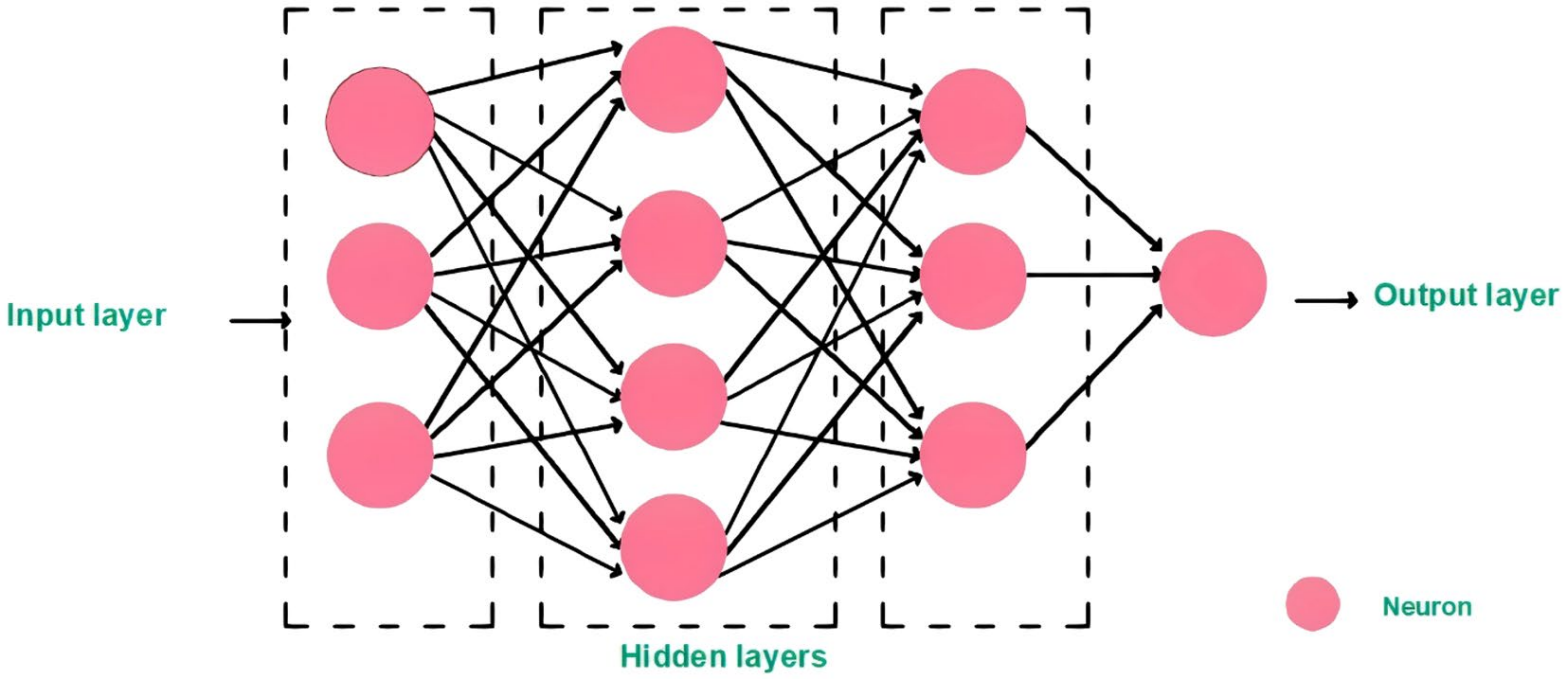
ANN layers.

As shown in Fig. 2, the standard structure of ANN consists of three layers:

- **Input layer**: It is a cluster of artificial neurons and its function is to receive the input data.
- **Hidden layers**: It is a cluster of artificial neurons and its function is computing the inputs.
- **Output layer**: It is a cluster of artificial neurons and its function is to deliver the outputs.

Each neuron receives signals from all neurons in the previous layer and each signal has a decimal weight. The sum of all input signals is applied to the neuron function then the result will be the neuron output weighted signal and it will move forward to all neurons in the next layer.

This is the standard design of ANN. ANN designer can control the direction of the signals and select the output signal destination^18^. Many researchers used ANN to represent the SOFC model and optimize its outputs. In^19^, ANN is used to calculate the SOFC-operated voltage and it consists of a single hidden layer with five neurons to compute the current, anode temperature, mass flow at the anode and cathode, and time. In^20^, ANN consists of a single input layer with 3 neurons which are the current, flow rate, and air temperature, and one hidden layer with 11 neurons to estimate the operating voltage as an output. On the other hand, ANN uses various optimization techniques such as genetic programming and, support vector machine to enhance SOFC performance, and a comparison between those techniques is presented in^21^.

### (ANFIS) mathematical demonstration

As mentioned earlier, the Sugeno method is used in the ANFIS technique. The following is the detailed explanation.

### Sugeno method

As mentioned in^19^ the mathematical Sugeno model is as follows:

- Assume that the fuzzy system has two inputs ${x}_{1}$ and ${x}_{2}$ as antecedents and one output ${y}_{i}$ As a consequence and $i=\text{1,2},\dots$ number of rule functions.
- Assume we have two rule functions:
  2 $$\text{Rule }1:\text{ if }{x}_{1}is {A}_{1}\text{and} {x}_{2} is {A}_{2}\text{ then }{y}_{1}=({b}_{1}{x}_{1}+{c}_{1}{x}_{2}+{d}_{1})$$
  3 $$\text{Rule }2:\text{ if }{x}_{1} is {A}_{2}\text{ and }{x}_{2} is {A}_{1}\text{ then }{y}_{2}=({b}_{2}{x}_{1}+{c}_{2}{x}_{2}+{d}_{2})$$

${A}_{1}$ and ${A}_{2}$ Are fuzzy sets for two different cases, e.g. Hot and cold.

${b}_{i}, {c}_{i}, and{ d}_{i}$ Are the constants established based on a piece of previous information or an opinion questionnaire by experts.

**Figure 3:**
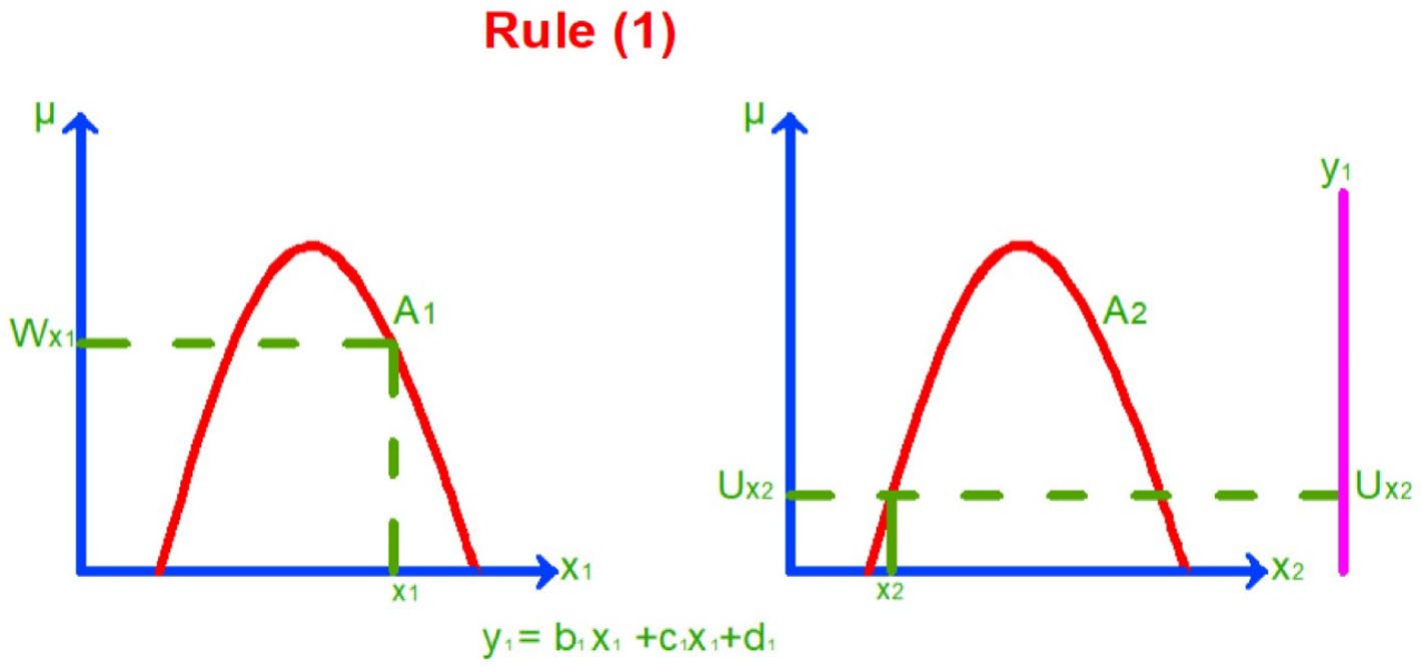
Membership value for inputs ${x}_{1}$ and ${x}_{2}$ according to rule (1).

**Figure 4:**
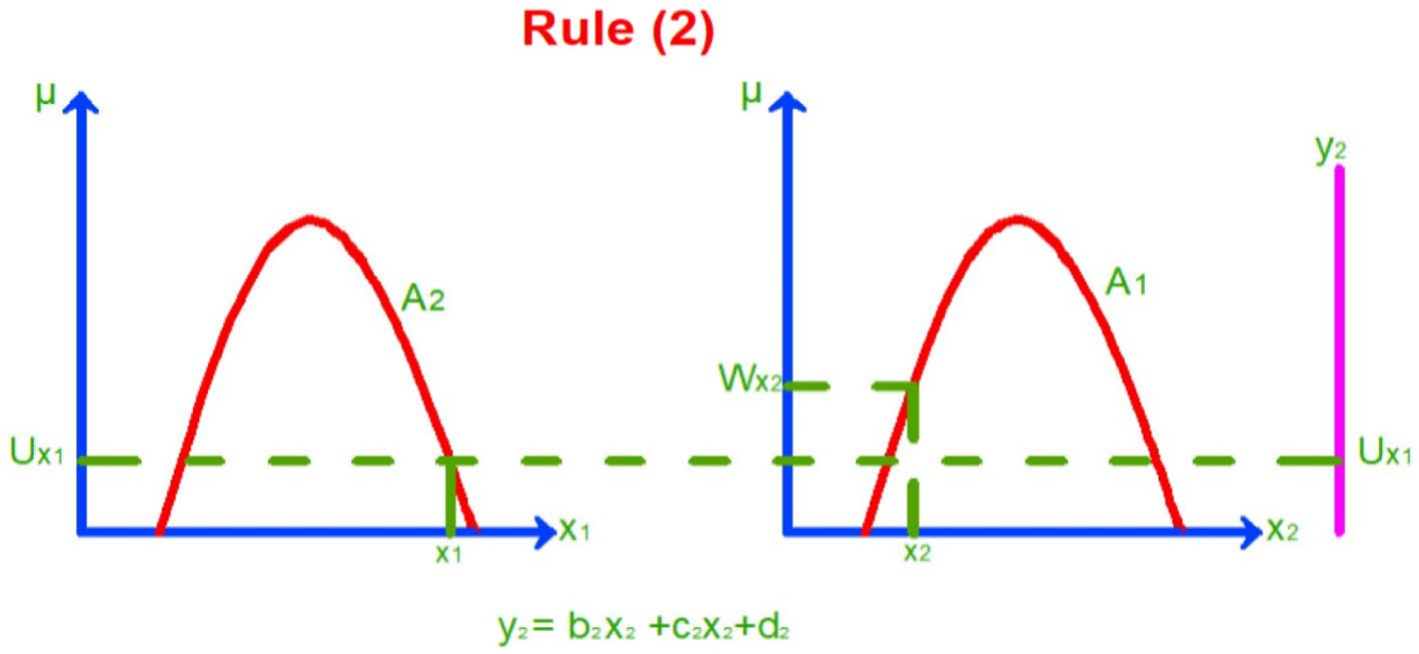
Membership value for inputs ${x}_{1}$ and ${x}_{2}$ according to rule (2).

- Then we plot the rule functions and determine the membership values of ${x}_{1}$ and ${x}_{2}$ as shown in Figs. 3 and 4.
- In the Sugeno method, the rule functions are a form of logic AND gates. According to the AND operator, the output is the minimum input. So, from the rule function and Fig. 3, the output will be as follows:
- Rule 1: when ${x}_{1} is {A}_{1}$, the membership is ${W}_{{X}_{1}}$ and when ${x}_{2 }is {A}_{2}$, the membership is ${U}_{{X}_{2}} {\text{where} U}_{{X}_{2}}$ has been chosen as an output because it is the minimum value due to the AND operator.
- Rule 2: when ${x}_{1} is {A}_{2}$, the membership is ${U}_{{X}_{1}}$ and when ${x}_{2} is {A}_{1}$, the membership is ${W}_{{X}_{2}}$ as shown in Fig. 4, ${U}_{{X}_{1}}$ Has been chosen as an output because it is the minimum value due to the AND operator.
- Then we solve the rule functions by Substitution crisp values ${x}_{1}$ and ${x}_{2}$ in the equations ${y}_{1}$ and ${y}_{2}$.
- Then we apply the results to the defuzzification function according to the Sugeno method to get the output as follows:
  4 $${y}^{*}= \frac{{U}_{{X}_{2}}{y}_{1}+ {U}_{{X}_{1}}{y}_{2}}{{U}_{{X}_{1}}+{U}_{{X}_{2}}}$$

So, ${y}^{*}$ Is the FIS output based on the Sugeno inference method.

### Fuzzy logic controller limitation

Based on the provided information above, it is clear that, if the experts set the wrong rule function, the result will be inaccurate and the controller will not be able to improve the performance. This means that FIS is not an expert and cannot be learned to find the appropriate rule function.

### The combination of ANN and FIS

The limitation of the FIS can be overcome using ANN, as ANN only requires inputs and outputs until they learn how to access these outputs by themselves.

**Figure 5:**
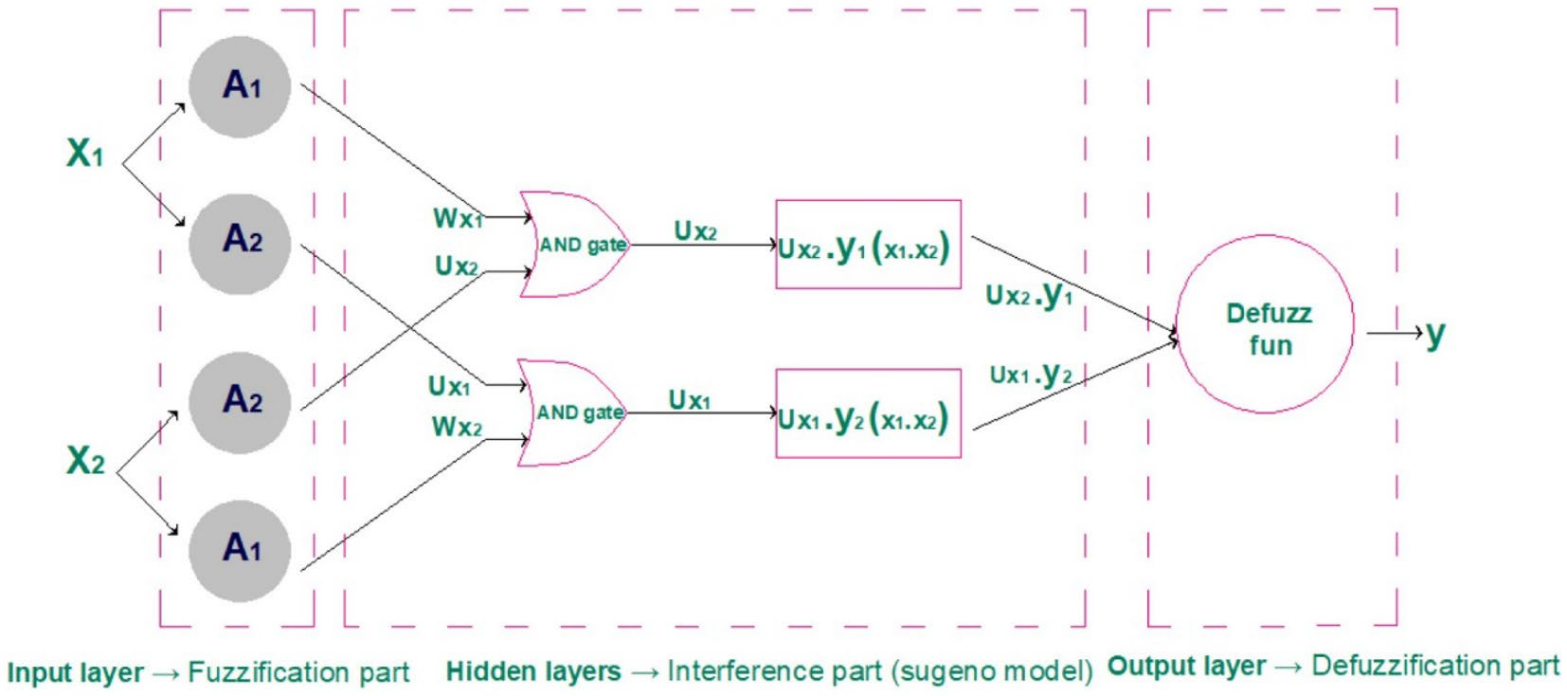
ANFIS architecture.

ANFIS technique is a developed AI and it is deduced by training the ANN to act as a fuzzy inference system to obtain the appropriate rule functions and assign the correct membership values that minimize the output function^18^. Assuming that only inputs $${(x}_{1}$$,$${x}_{2})$$ and one output $$({y}^{*})$$ are provided to the ANFIS, the ANN will be formed as shown in Fig. 5, and ANN learn to formulate the rule function as previously assumed in Eqs. (2) and (3).

In ANFIS, ANN layers represent FIS as follows:

Input layer $\to$ Fuzzification part based on rule functions.

Hidden layers $\to$ inference part (Sugeno model).

Output layer $\to$ Defuzzification part.

As shown in Fig. 5, the combination of ANN and the FIS is as follows:

The input layer has four neurons:

The first neuron function is to calculate the membership value when ${x}_{1} is {A}_{1}$ and the neuron output is ${W}_{{X}_{1}}$. The second neuron function is to calculate the membership value when ${x}_{1} is {A}_{2}$ and the neuron output is ${U}_{{X}_{1}}$. The third neuron function is to calculate the membership value when ${x}_{2 }is {A}_{2}$ and the neuron output is ${U}_{{X}_{2}}$. The fourth neuron function is to calculate the membership value when ${x}_{2 }is {A}_{1}$ and the neuron output is ${W}_{{X}_{2}}$.

Hidden layers consist of two layers:

The first layer consists of two similar neurons. These neurons have an AND operator to select the minimum input signal and its output ${U}_{{X}_{2}}$ and ${U}_{{X}_{1}}$ respectively. The second layer has two neurons in their function multiply input signal with ${y}_{1}({x}_{1},{x}_{2})$ in the first neuron and ${y}_{2}({x}_{1},{x}_{2})$ in the second neuron, and its outputs are ${U}_{{X}_{2}}.{y}_{1}({x}_{1},{x}_{2})$ and ${U}_{{X}_{1}}.{y}_{2}({x}_{1},{x}_{2})$ respectively.

Finally, the output layer consists of one neuron and its function is to compute defuzzification output by applying input signals to the Sugeno defuzzification function given in Eq. (4) and the neuron's output is ${y}^{*}$

### System under study

**Figure 6:**
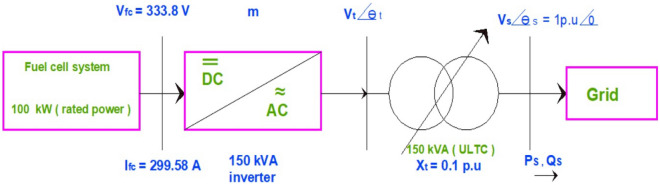
Under study grid-connected SOFC system with its rating values.

Figure 6 shows the grid-connected SOFC system under study. The system consists of the fuel cell system, under-load tap changer (ULTC), and an inverter. The inverter controls the voltage and frequency by adjusting the inverter modulation index ($$m).$$ The inverter is responsible for the synchronization with the grid with an acceptable voltage regulation $$of \pm 2\text{\%}$$ and frequency of about $$\pm 0.5\text{\%}.$$ the generated power ($${P}_{s}$$,$${Q}_{s}$$) is injected into the grid. The inverter consists of IGBT switches; therefore, it has a faster dynamic response than SOFC as its dynamic response depends on chemical reactions resulting after any change in the flow rate of hydrogen feeding it.

- The grid is modeled as an infinite bus bar with voltage $\left({V}_{s}\right)=1 p.u \text{and phase ang}le ({\theta }_{s})=0$
- The fuel cell system is modeled as a SOFC stack with operating voltage ($${V}_{fc})$$ and DC current ($${I}_{fc}$$).
- (ULTC) modeled as the transformer reactance $({X}_{t}$)
- The inverter is modeled as terminal voltage (${V}_{t}$ ), the inverter phase angel(${\theta }_{t}$), and modulation index ($m$)

### System analysis

In^11^, there are a detailed analysis of the fuel cell and all the equations that represent the grid–connected SOFC. In this paper, a background will be clarified about some of these equations to know the inputs and outputs that were used in the ANFIS controller. In SOFC, a volt is produced by energy resulting from chemical reactions inside the fuel cell, and this volt is calculated using the famous Nernst equation, as follows:

5 $${V}_{fc}={N}_{o}\left\{{E}_{o}+\frac{RT}{2F}In\left(\frac{{P}_{{H}_{2}}\sqrt{{P}_{{O}_{2}}}}{{P}_{{H}_{2}O}}\right)\right\}-{I}_{fc}.r$$

where ${V}_{fc}$ is the fuel cell DC voltage, ${\varvec{r}}$ Is the FC stack resistance in the ohm unit, $({{\varvec{P}}}_{{{\varvec{H}}}_{2}})$ is the partial pressure of the hydrogen in atm, ${({\varvec{P}}}_{{{\varvec{H}}}_{2}{\varvec{O}} })$ is the partial pressure of the water in atm, ${({\varvec{P}}}_{{{\varvec{O}}}_{2} })$ is the partial pressure of oxygen in atm, ${{\varvec{N}}}_{{\varvec{o}}}$ is the number of fuel cells connected in series forming a fuel cell stack, $R$ is the ideal gas constant = 8.314 (J/mol/K), $\text{T}$ is the temperature in kelvin, $\text{F}$ is Faraday’s constant = 96,487 (C/mol), and ${I}_{fc}$ is the fuel cell current in Ampere.

The generated DC power from the SOFC is given by:

6 $${P}_{dc}={V}_{fc}{I}_{fc}$$

The electric current produced from the fuel cell depends on the load and the flow rate of hydrogen entering the fuel cell $({q}_{{H}_{2}}^{in})$, the ${U}_{f}$ cannot be increased or decreased from specific values so that the fuel cell is not exposed to damage. This restriction can be formulated in the following function:

7 $$0.8\le {U}_{f}\le 0.9$$

On this basis, the fuel cell control strategies have been developed and the dynamic equations that describe these strategies are as follows:

8a $$\frac{d{I}_{fc}}{dt}=\frac{1}{{\tau }_{e} }\left(\frac{{P}_{ref}}{{V}_{fc}}-{I}_{fc}\right)\text{ For constant power control strategy}$$

8b $$\frac{d{I}_{fc}}{dt}=\frac{1}{{\tau }_{e} }\left(\frac{{P}_{ref}}{{V}_{{fc}_{o}}}-{I}_{fc}\right)\text{ For constant current control strategy}$$

${P}_{ref}$ Is the reference power, ${\tau }_{e}$ is the electrical response time.

${V}_{{fc}_{o}}$ Is referring to using a constant fuel cell voltage value in the grid-connected SOFC simulated model.

The fuel cell produces DC voltage. Therefore, it is necessary to connect SOFC to the grid through an inverter which is suitable for the ratings of the fuel cell. The inverter output voltage ($${V}_{t})$$ and its phase angle ($${\theta }_{t}$$ ) are given by:

9 $${V}_{t}=0.6124{mV}_{fc}$$

10 $${\theta }_{t}={\theta }_{s}+ {\text{sin}}^{-1}\left[\frac{{I}_{fc} {X}_{t}}{0.6124{mV}_{s}}\right]$$

As assumed in^11^, the inverter/transformer losses are neglected. Consequently, the injected real power and reactive power to the grid are calculated as follows:

11 $${P}_{s}=\frac{{V}_{s} {V}_{t}}{{X}_{t}}\text{sin}\left({\theta }_{t}- {\theta }_{s}\right)={V}_{fc}.{I}_{fc}$$

12 $${Q}_{s}=\frac{{V}_{s} {V}_{t}}{{X}_{t}}\text{cos}\left({\theta }_{t}- {\theta }_{s}\right)-\frac{{V}_{s}^{2}}{{X}_{t}}$$

The inverter control process is described as follows:

13 $$\frac{m}{{V}_{ref}-{V}_{t}}=\frac{{K}_{m}}{1+s{\tau }_{m}}\text{ And }{V}_{ref}=1.05 p.u$$

${K}_{m}$ Is the gain of the voltage control loop,${V}_{ref}$ is the reference voltage to the voltage controller, and ${\tau }_{m}$ is the time constant of the voltage control loop.

From equations (8a) and (8b), it is clear that to improve the performance of the control circuit that was designed based on these equations to control the SOFC output current and thus control the DC power produced from it, the ANFIS controller is added to this control circuit as follows:

14 $$\text{ANFIS input }= \left(\frac{{P}_{ref}}{V}-{I}_{fc}\right)$$

15 $$\text{ANFIS output }= \frac{dI}{dt}.{\tau }_{e}$$

From equations (9), (11), and (12), we conclude that if the output of the inverter is controlled, then the injected power to the grid is controlled, so equation (13) is used to design the control circuit for the grid’s injected power. Therefore, the ANFIS controller has been added to this circuit as follows:

16 $$\text{ANFIS input }=\left[{K}_{m}\left({V}_{ref}-{V}_{t}\right)\right]-m$$

17 $$\text{ANFIS output }= \frac{dm}{dt}.{\tau }_{m}$$

As mentioned above, the SOFC was fully modeled and described in^11^. So, other equations describe the dynamic response inside the SOFC to determine the pressure and temperature inside it, and they are as follows:

18 $${P}_{{H}_{2}} {V}_{an}= {n}_{{H}_{2 }}RT$$

${V}_{an}$ Is the volume of the anode, $R$ is the ideal gas constant, ${n}_{{H}_{2}}$ and is the moles of hydrogen.

19 $$\frac{{dP_{{H_{2} }} }}{dt} = \frac{1}{{\tau_{H} }}{ }\left\{ {\frac{1}{{K_{H2} }}\left( {q_{{H_{2} }}^{r} - 2K_{r} I} \right) - P_{{H_{2} }} } \right\}$$

${\tau }_{H}$: The hydrogen flow response time.

20 $${K}_{r}=\frac{{N}_{o}}{4F}$$

21 $$\frac{{dP}_{{H}_{2}O }}{dt}=\frac{1}{{\tau }_{{H}_{2}O}}\left\{\frac{{2K}_{r}I}{{K}_{{H}_{2}O}}-{P}_{{H}_{2}O}\right\}$$

${\tau }_{{H}_{2}O}$: The water flow response time.

22 $$\frac{{dP}_{{O}_{2} }}{dt}=\frac{1}{{\tau }_{{O}_{2}}}\left\{\frac{1}{{K}_{{O}_{2}}}\left(\frac{{q}_{{H}_{2}}^{in}}{{r}_{HO}}-{K}_{r}I\right)-{P}_{{O}_{2}}\right\}$$

${\tau }_{{O}_{2}}$: The oxygen flow response time.

In this research, the ANFIS controller was not used to control any of the parameters that represent the dynamic response within the SOFC such as ${{\varvec{P}}}_{{{\varvec{H}}}_{2}}$,${{\varvec{P}}}_{{{\varvec{O}}}_{2}}$, and ${{\varvec{P}}}_{{{\varvec{H}}}_{2}O},$ but as it is clear from Eqs. 14, 15, 16, and 17, the ANFIS controller was used to control the SOFC DC output and the inverter AC output, so it is used as a DC/AC controller and not as a SOFC controller.

As noticed in the ANFIS controllers which are added to the control circuits, in each chosen control circuit, the ANFIS controller has one input and one output, and the number of the rule functions can be chosen by the ANFIS designer in the MATLAB/SIMULINK. When the chosen number of the rule functions is increased, the accuracy of the results increases, but this increase is accompanied by a long time in ANFIS training and extracting the results. Therefore, the number of ten rule functions was chosen to increase the result's accuracy and reduce the time of the ANFIS training and extracting the results.

**Figure 7:**
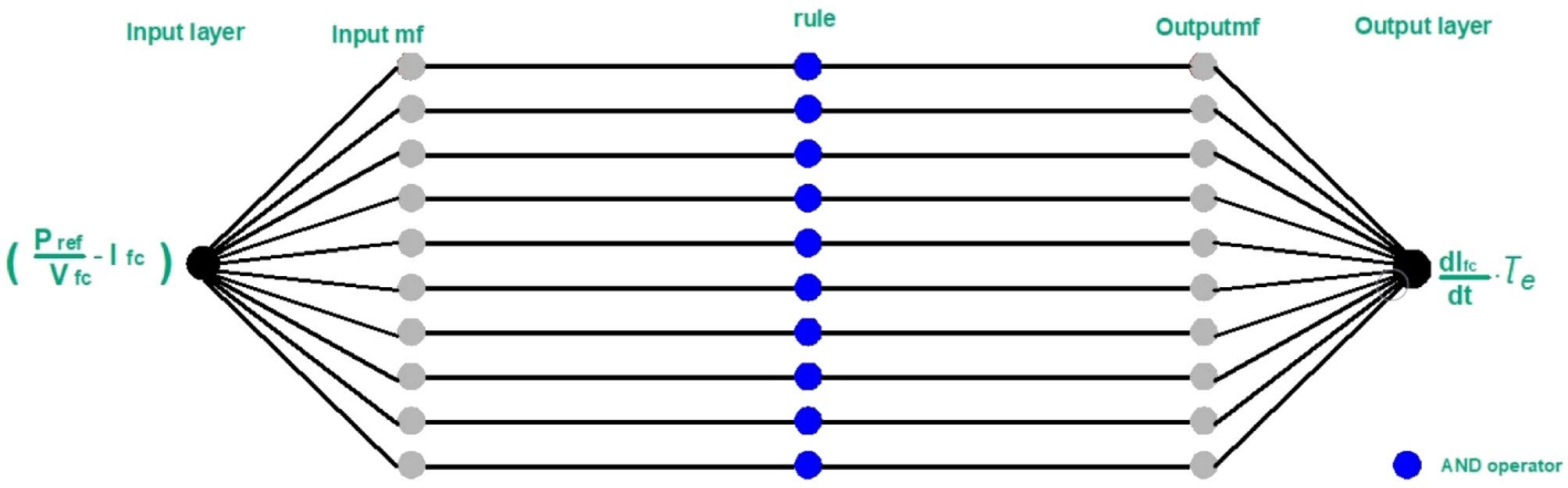
The added one input/one output ANFIS controller to the DC power control circuit.

**Figure 8:**
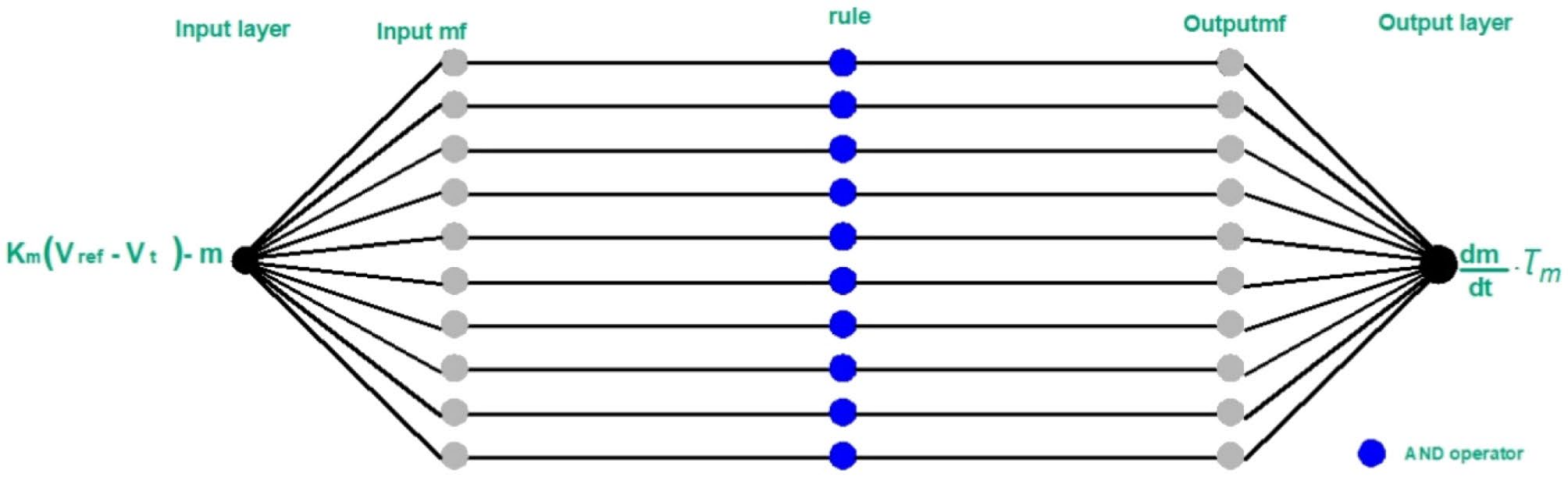
The added one input/one output ANFIS controller to the AC power control circuit.

In Figs. 7 and 8, the ANFIS structure that is used in grid-connected SOFC. The role of the ANFIS controller is to minimize the deviation of system parameter values from its steady-state values before the applied disturbance.

### Performance of SOFC as affected by scheduled load disconnecting and controlled by ANFIS

As mentioned earlier in^6^, $100 \text{kW}$ SOFC stack grid-connected was modeled and controlled with a constant current control strategy. The SOFC model parameters are given in^7^. Using MATLAB SIMULINK to simulate the scheduled load disconnecting and test grid-connected SOFC’s dynamic response, the model was subjected to a 10% step-down in reference power $({P}_{ref})$ at the fifth second after starting the system and the results were extracted to show the dynamic performance with and without adding ANFIS controller. Please notice that: In the results figures, anfis refers to results by adding ANFIS to the model and c.c refers to results with the constant current control strategy without adding ANFIS to the model.

**Figure 9:**
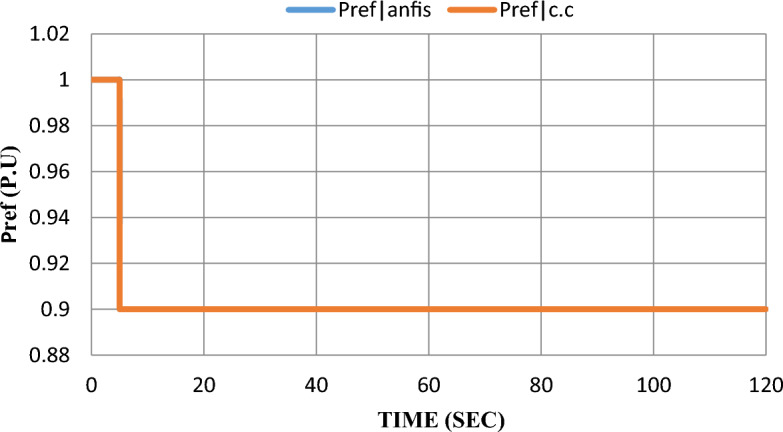
Applied disturbance; 10% decrease in the reference power.

As shown in Fig. 9, the applied disturbance to the grid-connected SOFC is a 10% decrease in the reference power.

**Figure 10:**
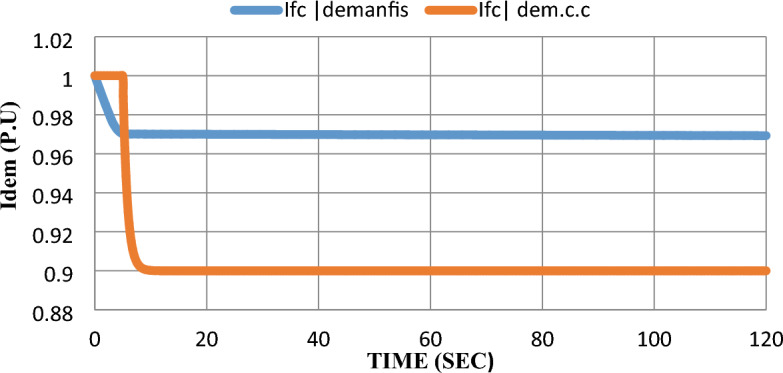
Unmodified fuel cell dc demand current response after the disturbance.

As shown in Fig. 10, using the ANFIS technique enhances the performance of the SOFC model under disturbance whereas the SOFC demand current ($${I}_{fc dem})$$ steady-state error was decreased from 9 to 3%.

**Figure 11:**
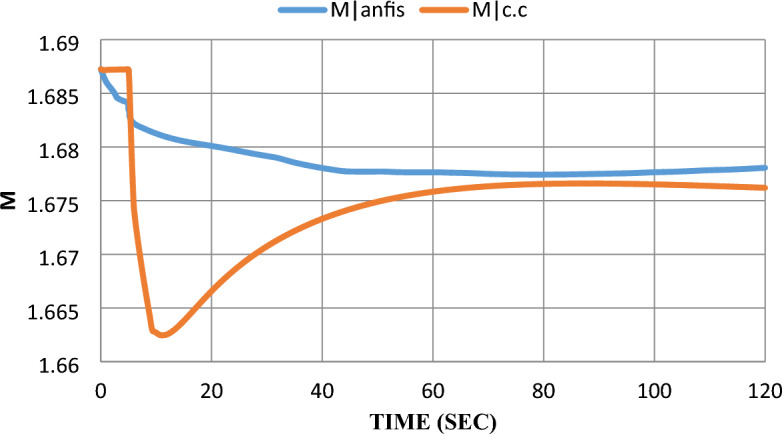
Modulation index response after the disturbance.

As shown in Fig. 11, the ANFIS technique succeeded in eliminating the former 1% undershoot in the inverter modulation index ($m$) and the steady-state error was decreased from 2% to 0.3%.

**Figure 12:**
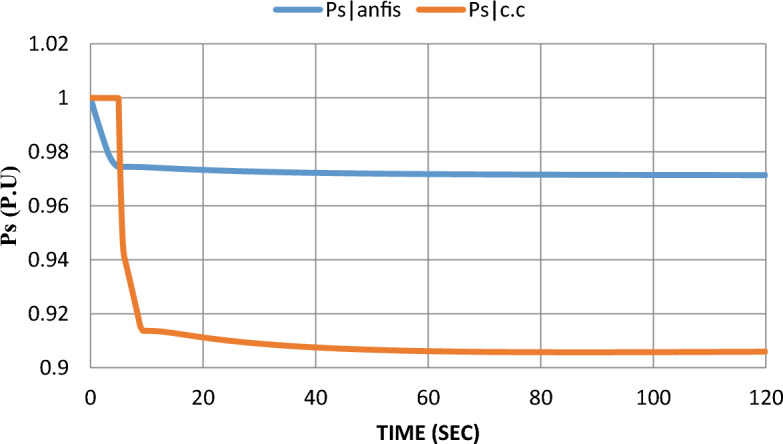
Converter active power response after the disturbance.

As shown in Fig. 12, using the ANFIS technique enhances the performance of the SOFC model under disturbance whereas the injected active power to the grid ($${P}_{s})$$ steady-state error was decreased from 9 to 3%, and settling time was enhanced from 9 to 5 s.

**Figure 13:**
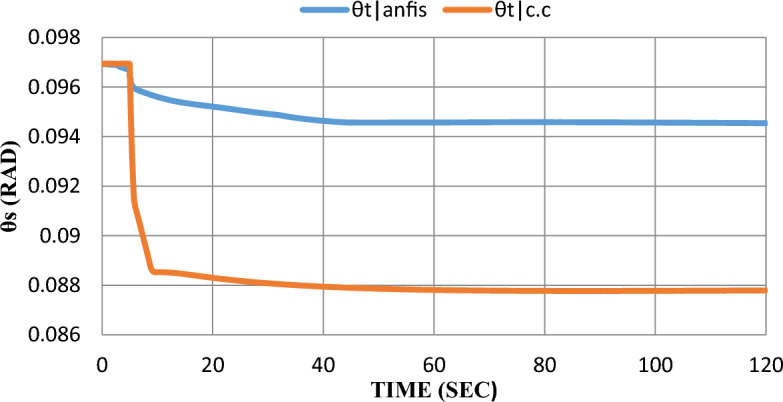
Phase angle response after the disturbance.

As shown in Fig. 13, the dynamic response of the phase angle (${\theta }_{t}$) was enhanced by adding the ANFIS technique to the SOFC model. Steady-state error was decreased from 9 to 2%.

**Figure 14:**
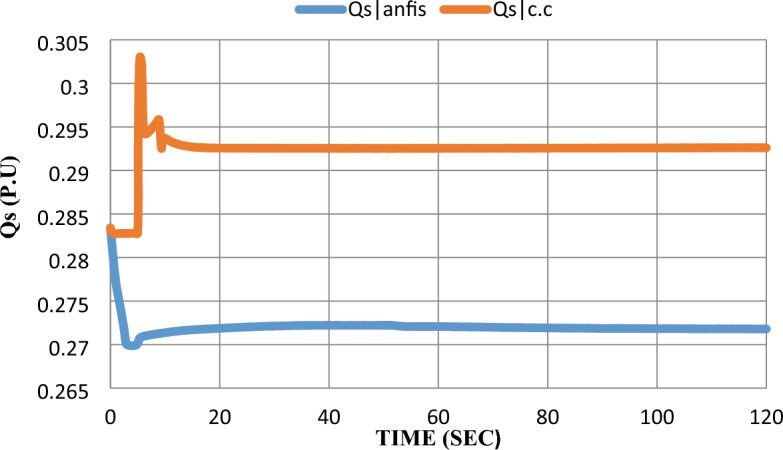
Reactive power response after the disturbance.

As shown in Fig. 14, in the constant current control strategy, the injected reactive power to the grid ($${Q}_{s})$$ had a 7% overshoot after the disturbance was subjected to the SOFC model. Adding the ANFIS technique eliminates the overshoot and keeps a steady state error of 3%, which is the same with and without adding the ANFIS.

**Figure 15:**
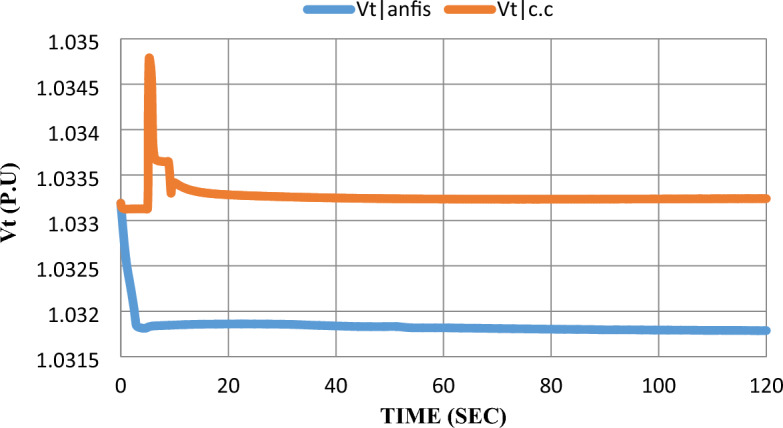
Converter terminal voltage response after the disturbance.

As shown in Fig. 15, in the constant current control strategy dynamic response, the voltage on the inverter terminals (${V}_{t}$) had a 2% overshoot after the disturbance was subjected to the SOFC model. Adding the ANFIS controller to the model eliminates the overshoot and enhances settling time from 5 to 3 s.

**Figure 16:**
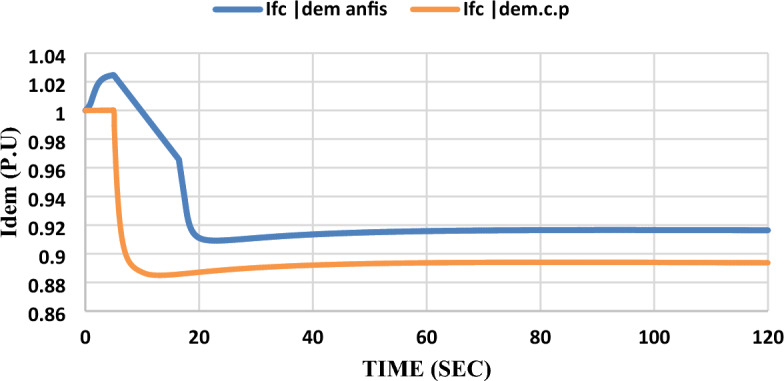
Unmodified fuel cell dc demand current response after the disturbance.

**Figure 17:**
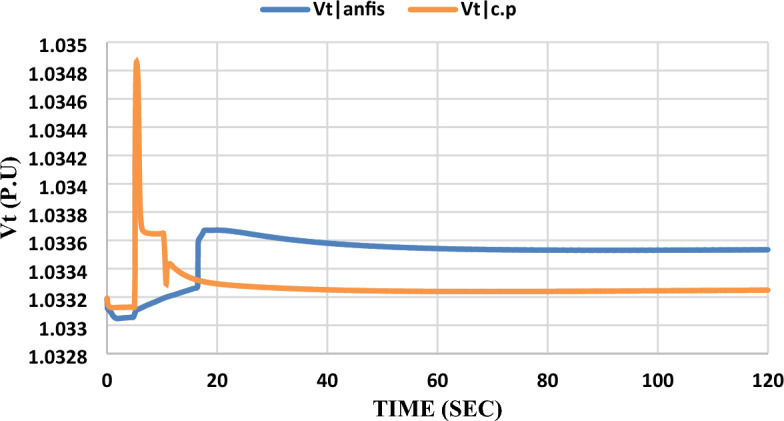
Converter terminal voltage response after the disturbance.

As shown in Figs. 16 and 17, the results indicate that when adding the ANFIS controller to the grid-connected SOFC, which was previously controlled by a constant power control strategy and subjected to the same disturbance, it was found that its dynamic response to the same disturbance is not better than the model that was controlled by the constant current control strategy and the ANFIS controller was added to it in terms of maximum overshoot, steady state error, and settling time. Please notice that: In the results figures, anfis refers to results by adding ANFIS to the model and c.p refers to results with the constant power control strategy without adding ANFIS to the model.

As shown in Fig. 16, the ANFIS controller succeeded in decreasing the SOFC demand current ($${I}_{fc dem})$$ steady-state error from 11 to 9% but the settling time was increased from 10 to 20 s, and it caused an overshoot with 3%.

As shown in Fig. 17, the ANFIS controller succeeded in eliminating the inverter terminal voltage (${V}_{t}$) overshoot but the steady state error was increased from 0.02% to 0.06%.

As shown in the extracted results, the ANFIS technique has proven that it can enhance grid-connected SOFC which was previously controlled by a constant current control strategy after being subjected to disturbance by learning from SOFC model data and its performance before adding ANFIS to the SOFC model. ANFIS decreased steady-state error for all parameters and eliminated overshoot-undershoot for ${V}_{t}$, Qs, and $m$. Settling time has been reduced for ${P}_{s}$ and ${V}_{t}$ by 4 s and 2 s respectively.

It also became clear from the results that the ANFIS controller succeeded in reducing the deviation from the steady-state value more than the other control strategy, whether by reducing over-shoot steady-state error or settling time, when ANFIS controller outputs were chosen, it had to be changed several times until the ANFIS settles on the best results that it can reach, considering to the number of rule functions that are chosen to reach these results.

In other research such as^9^ and^10^, regardless of the control strategies, the PI controller remains the main control type, and in^6^, there are two control strategies, but the main control type is state-feedback controllers, in these two control types, the dynamic performance enhancement techniques are limited, such as PI controller tuning, but the ANFIS controller can start improving the dynamic performance from where the other control types ended due to its ability to learn the way any system works and its flexibility in improving results, where the ANFIS designer can reach the best results by doing Many ANFIS training with different number of rule functions.

So, the results prove that the ANFIS controller can enhance the performance of any system which is controlled by any control type.

## Conclusion

Artificial neural networks (ANN) and adaptive neuro-fuzzy inference system (ANFIS) techniques are used to investigate the performance of grid-connected (SOFC) operations. The combined ANN and FIS are used to predict performance parameters and dynamic response of stack current, voltage, and power through a system under study using a constant current control strategy. Using MATLAB/SIMULINK, the ANFIS controller was added to the grid-connected SOFC model under the control of a constant current control strategy so that the SOFC model could be learned and its dynamic performance is enhanced and tested through the application of a scheduled load disconnecting scheme.

The results showed that the ANFIS controller can enhance the SOFC model dynamic response. This is demonstrated in the obtained results that assure minimum steady-state error, less overshoot-undershoot $,$ and fast settling time in all the obtained dynamic time responses of the grid-connected SOFC studied system under constant control.

## Data Availability

The datasets used and/or analyzed during the current study are available from the corresponding author upon request.

## Abbreviations

SOFC: Solid oxide fuel cell
ANFIS: Adaptive neuro-fuzzy inference system
GHG: Greenhouse gases
ICE: Internal combustion engines
ILC: Interlinking converter
PCC: Point of common coupling
SMC: Sliding Mode Controller
NN: Neural networks
FL: Fuzzy logic
RES: Renewable energy sources
FC: Fuel cell
AFC: Alkaline fuel cell
MCFC: Molten-carbonate fuel cell
AI: Artificial intelligence
ML: Machine learning
ANN: Artificial neural networks
FIS: Fuzzy inference system
ULTC: Under-load tap changer
